# Supplementary material for: Diurnal variations of amplitude of accommodation in different age groups
Source: PLoS One. 2019 Nov 26;14(11):e0225754. doi: 10.1371/journal.pone.0225754 (PMC6879161; doi:10.1371/journal.pone.0225754)
Supplement: Attachment — Submitted filename: Response_to_Reviewers.docx [file pone.0225754.s002.docx]

Journal Requirements:

(Requirements and Reviewer’s comments are shown in *Italic,* line numbers pointed are number of revised manuscript)

*When submitting your revision, we need you to address these additional requirements.*

*Please ensure that your manuscript meets PLOS ONE's style requirements, including those for file naming. The PLOS ONE style templates can be found at http://www.journals.plos.org/plosone/s/file?id=wjVg/PLOSOne_formatting_sample_main_body.pdf and http://www.journals.plos.org/plosone/s/file?id=ba62/PLOSOne_formatting_sample_title_authors_affiliations.pdf*

**Response to Journal requirements (RJ1):** We have verified and revised our revised manuscript to ensure that it meets the journal requirements regarding the ‘Manuscript body formatting guidelines’ and the ‘Title, author, affiliations formatting guidelines’.

*1. Please provide additional details regarding participant consent. In the Methods section, please ensure that you have specified (1) whether consent was informed and (2) what type you obtained (for instance, written or verbal). Please state in your methods section whether you obtained consent from parents or guardians of the minors included in the study or whether the research ethics committee or IRB approved the lack of parent or guardian consent.*

**RJ2**: We thank the editor for the pertinent comments. As requested, we added as follows: “…and written informed consent was obtained from adult participants, and from parents or guardians of minors” has been included in the Materials and methods section, Subjects subsection, page 6, lines 117-119.

Additional Editor Comments:

Additional Editor Comments (if provided):

*This study involves psychology/behavioral research conducted using the online platform Amazon Mechanical Turk. Because workers on these platforms are often experienced in participating in such studies or may be motivated to participate when they should not, please confirm that you sufficiently addressed the issues of non-naivety and trustworthiness.*
**Response to Additional Editor Comments (RE1)**: Thank you for providing a psychology/behavioral research approach conducted using the online platform Amazon Mechanical Turk (AMT). However, we do not have much information about the AMT concepts yet and we require additional effort to be able to utilize it. We believe that our study should be evaluated directly by professional examiners, rather than as a survey study that is based on the use of questionnaires.

[Note: HTML markup is below. Please do not edit.]

Comments to the author:

Reviewers' comments:

Reviewer's Responses to Questions

**Comments to the Author**

1. Is the manuscript technically sound, and do the data support the conclusions?

The manuscript must describe a technically sound piece of scientific research with data that supports the conclusions. Experiments must have been conducted rigorously, with appropriate controls, replication, and sample sizes. The conclusions must be drawn appropriately based on the data presented.

Reviewer #1: No
Reviewer #2: Yes

**Authors’ Response**: We have replied and partly revised the manuscript as stated in response to the reviewer #1’s comments (R1R#1, R2R#1, R3R#, R5R#1, R7R#1, and R8R#1).

2. Has the statistical analysis been performed appropriately and rigorously?

Reviewer #1: No
Reviewer #2: Yes

**Authors’ Response**: We have also noted in R9R#1 and R10R#1 in response to the reviewer #1’s comments regarding issues such as ‘not reliable and a high variability’.

3. Have the authors made all data underlying the findings in their manuscript fully available?

The [PLOS Data policy](http://www.plosone.org/static/policies.action#sharing) requires authors to make all data underlying the findings described in their manuscript fully available without restriction, with rare exception (please refer to the Data Availability Statement in the manuscript PDF file). The data should be provided as part of the manuscript or its supporting information, or deposited to a public repository. For example, in addition to summary statistics, the data points behind means, medians and variance measures should be available. If there are restrictions on publicly sharing data—e.g. participant privacy or use of data from a third party—those must be specified.

Reviewer #1: Yes
Reviewer #2: No

**Authors’ Response**: We have added a statement regarding this in R1R#2 and R2R#2.

4. Is the manuscript presented in an intelligible fashion and written in standard English?

PLOS ONE does not copyedit accepted manuscripts, so the language in submitted articles must be clear, correct, and unambiguous. Any typographical or grammatical errors should be corrected at revision, so please note any specific errors here.

Reviewer #1: Yes
Reviewer #2: Yes

**Authors’ Response**: Thank you for the positive comments. The revised manuscript has also been re-edited by a native English speaker.

5. Review Comments to the Author

Please use the space provided to explain your answers to the questions above. You may also include additional comments for the author, including concerns about dual publication, research ethics, or publication ethics. (Please upload your review as an attachment if it exceeds 20,000 characters)

Reviewer #1: INTRODUCTION

*- Page 3 line 53-55: Why AA is important to diagnosis either accommodative insufficiency or the accommodative excess?*

**Response 1 to Reviewer #1 (R1R#1):** We thank the reviewer for the valuable comments. As shown in reference ‘[7]’, accommodative insufficiency occurs when the AA is lower than expected for the patient's age. Accommodative excess is a condition in which the accommodative system fails to respond to any stimulus, although AA is a normal amplitude. Accommodative disorders differ depending on whether AA is normal or abnormal. Therefore, AA should be evaluated as a differential diagnosis of accommodative disorders. Therefore, ‘the diagnosis of accommodative disorders’ was replaced with ‘differential diagnosis of accommodative disorders’ in the Introduction section, page 3, line 54.

*- Page 3 line 59-64: Why knowing all the limitations about subjective methods to measure the AA, you chosen the Push up.*

**R2R#1**: We thank the reviewer for the pertinent comment. It is well-known that there are advantages and disadvantages to various subjective methods used for measuring AA. The push-up method is a widely used method among such methods despite having these limitations. The push-up method is quick and easy to perform and an assessment of the near point of clear vision, relates to the typical symptom reported by early presbyopes [R1], although the push-up method tend to slightly overestimate the AA because of the relative distance magnification of the letter target. Therefore, we have included the following in the Introduction section “The push-up method among subjective methods, has the disadvantage of slightly overestimating the AA because of the relative distance magnification of the letter target, but this method is quick and easy to perform” as a limitation and merit of the push-up method in lines 63-66.

Reference:

[R1] Elliott DB. Clinical procedures in primary eye care. 3rd ed. Edinburgh: Butterworth-Heinemann; 2007. pp. 191-194.

*- Page 4 line 71-72: “In clinical practice, however, it is more important to assess AA based on the time of the day or the patient’s age.” Why or how you support this affirmation, indeed, in real world few times a subject has to do a lot of accommodation.*

**R3R#1**: The reasons why we have to assess AA based on the time of the day or the patient’s age are as follows: 1) Patients may complain about blurring after sustained reading or at the end of the day, and accommodative disorders may be better revealed at the end of the day when fatigue is more likely to occur, as shown in reference ‘[7]’; 2) The AA related to presbyopes as noted in reference ‘[16]’ reveals differences more clearly according to age and the measurement points, during the day; and 3) We directly experienced the process of prescriptions for spectacles in clinical practice, and patients with accommodative insufficiency have lower AA at the end of the day than in the morning. The references regarding the above statements were included in the Introduction section, page 4, line 75.

*- Page 4, lines 78-85: According to that paragraph, I think, it would be better to measure the accommodative response (lag) or the accommodative facility.*

**R4R#1**: We thank the reviewer for providing that astute point of view. We partly agree with the reviewer’s comments. The accommodative response or accommodative facility should be included in the assessment of accommodative disorders. According to a reference ‘[8]’, in addition, indirect measures refer to tests such as the negative relative accommodation (NRA) and the positive relative accommodation (PRA) that are generally thought of as tests of accommodative function. In clinical practice, many authors have reported the clinical significance of testing accommodative response and facility as well as amplitude [R2]. Accommodative lag measures accommodative response to an accommodative stimulus and accommodative facility measures accommodative stamina as the speed of accommodative responsiveness. The tests are useful in the case of a person who may experience asthenopic symptoms and have other accommodative disorders even when the accommodative amplitude is normal [R3, R4]. However, accommodative lag and accommodative facility is related to binocular anomalies besides accommodative disorders. Both testing do not evaluate all accommodative functions. We would like to emphasize that our study was focused on AA. The traditional evaluation of accommodative function involves the measurement of the amplitude of accommodation using the push-up method.

Reference:

[R2] Scheiman M, Herzberg H, Frantz K, Margolies M. Normative study of accommodative facility in elementary schoolchildren. Am J Optom Physiol Opt. 1988; 65:127-134.

[R3] Hennessey D, Iosue RA, Rouse MW. Relation of symptoms to accommodative infacility of school-aged children. Am J Optom Physiol Opt. 1984; 61:177-183.

[R4] Wick W, Hall P. Relation among accommodative facility, lag, and amplitude in elementary school children. Am J Optom Physiol Opt. 1987; 61:177-183.

*- I cannot see any cause that support to perform that study.*

**R5R#1**: We thank the reviewer for the valuable and constructive comments. To make our study intention clear, as already stated in the ‘Introduction’, 1) The AA is an important test in evaluating accommodative disorders; 2) AA depends on age; 3) The push-up method is routinely used to measure the AA; and 4) As in R3R#1 above, the AA is likely to vary during the day. For these reasons, we conducted a study on the ‘Diurnal variations of amplitude of accommodation in different age groups’.

*- why you considered that age could be an important variability factor in the daily changes of the AA?*
**R6R#1**: Our response to this question is the same as above: R5R#1.

RESULTS:

*- Page 9, lines 201-207: I could not understand which increases or decreases you say.*

**R7R#1**: ‘increases and decreases’ have been replaced with ‘increases versus decreases’ in line 210, 212, and 214.

*- Figure 3: It is blur and I could not see it.*

**R8R#1**: Figure 3 has been revised for easy reading.

*- Page 10-11: The analysis of Table 3 is not clear, you say, "Regarding detection of differences (> 1.50 D) among AA values measured in each session" i.e., within the three times that you measured the AA in each S1, S2 etc, or the differences between S1, S2 etc; this is important, because in the first case, you are showing that the test used is not reliable (high AUC for adolescent and twenties) and the changes in the AA could be because a high variability by the Push up test.*
**R9R#1**: We thank the reviewer for the detailed comments on our work. We partly agree with the reviewer’s question on the reliability (maybe when comparing with other methods) of the push-up method. In summary, on the push-up method, its problems relates to the overestimation of the AA because of the relative magnification as noted in reference ‘[8]’. To compensate for the overestimation, the most commonly used alternative (modified push-up) involves using increasing amounts of minus spherical lens power until the distance vision blurs [R1]. However, the push-up method as a traditional method is faster and more widely used than other methods as noted in reference ‘[29]’. In comparing the measurement techniques for AA in reference ‘[28]’, the quick and easy assessment of the AA using the push-up and the push-down methods were compared to other methods (minus lens method, modified push-up method), and the perfect agreement obtained between these two methods can further emphasize their use as a routine procedure in the clinic, especially if a combination of the two techniques is used to offset their slight over- and underestimation.

The AA was measured using the push-up method in several studies, as shown in Table R1. Variability based on the mean and SD showed a tendency of decrease with age. These variabilities may be similar with our study findings. Although the measuring conditions such as the methodological differences affect the reliability of the AA, the ROC curve analysis based on the data (18 times for each individual person) that was obtained from the measurement carried out 3 times per person in 6 sessions per day would be meaningful. Our results showed significant or nonsignificant differences between sessions.

Table R1. Mean and standard deviation (SD) of the AA measured using the push-up method in several studies

|  | Mean ± SD | Subject | Age |
| --- | --- | --- | --- |
| Koslowe et al. [26] | 13.16 ± 2.69 D  11.10 ± 3.25 D | 30  25 | 13-20  21-35 |
| Momeni-Moghaddam et al. [28] | 11.21 ± 1.85 D | 52 students | 21.2 ± 1.6 |
| Mathebula et al. [29] | 10.23 ± 1.67 D | 45 optometry students | 21-27 |
| Anderson and Stuebing [R5] | 13.16 ± 3.45 D  10.57 ± 3.51 D  9.41 ± 2.01 D  8.45 ± 2.24 D  2.92 ± 1.98 D  0.47 ± 1.05 D | 22  26  23  25  25  5 | 11-15  16-20  21-25  26-30  41-45  46-50 |

We believe that a high variability and a low reliability do not account for all of the changes between sessions. In our study, it may be recalled that the value of significant difference between sessions was set at over 1.50 D.

Reference:

[R5] Anderson HA, Stuebing KK. Subjective versus objective accommodative amplitude: preschool to presbyopia. Optom Vis Sci. 2014; 91(11):1290-1301.

DISCUSSION

*- Page 16, lines 352-360: “significant diurnal changes in AA were observed between sessions measured at two-hour intervals from 9:00 a.m. to 8:00 p.m. and the changes were greater in younger age groups” I do not agree with this conclusion, because the large variability within each session could cause that differences, but no a really change in the amplitude of accommodation.*

**R10R#1**: We thank the reviewer for the valuable and rigorous review and comments, and for devoting your time to the review of our manuscript. However, we do not fully agree with the reviewer’s comments. We all feel discouraged when our study has a weakness: i.e. not reliable, or a high variability. Our study started from the possibility of subjective symptoms and fluctuations in the AA, experienced in the clinical field as indicated in the statement above in R3R#1. Although the push-up method has issues with reliability or variability, it is the commonest and simplest clinical technique to be used to measure amplitude of accommodation as stated in the general method for the clinical measurement of AA.

In our study, diurnal changes in each individual under the clinical measurement of AA conditions were distributed from 0.00 D to 6.67 D with 1.93 ± 1.41 D as the mean ± SD. These results suggest that diurnal changes in AA exist.

We hope that the review of our manuscript based on the reviewer’s comments has provided us with the chance to satisfactorily improve our manuscript.

*Reviewer #2: As stated by the authors in the introduction section, pupil diameter is important in the accommodation amplitude measurement. Have pupil diameters of the participants been measured? If yes, I suggest you give us some information.*

**R1R#2**: We thank the reviewer for the valuable comments suggested from a different point of view. The accommodation in response to focusing on an object comprises of coordinated changes in the lens and the pupil size. Our study focused on the diurnal change to the accommodative amplitude. There were no data on the pupil size. In a recent study by Suryakumar and Allison [R6] related to the pupil, the finding that accommodation and pupil responses increased with disparity regardless of the sign of retinal disparity suggests that these responses were driven by apparent depth rather than shifts in the mean simulated distance of the stimulus.

Reference:

[R6] Suryakumar R, Allison R. Accommodation and pupil responses to random-dot stereograms. J Optom. 2016; 9(1):40–46.

*I also wondered if the results were different between male and female participants. The gender of the participants is not present in the Excel file. In general, I must say that I like the article.*

**R2R#2**: We thank the reviewer for the relevant comment. We did not present the data on gender in the file due to the many missing gender data. As shown in a result in reference ‘[34]’, ‘Males had insignificantly higher mean AA than the age-matched females’. In Ovenseri-Ogbomo et al.’s study [R7], ‘gender difference in mean AA was statistically significant.’ We could not deduce or identify whether there was a difference between genders in our study. We seek for your understanding regarding the fact that we did not provide an answer to justify the role of gender.

 Reference:

 [R7] Ovenseri-Ogbomo GO, Kudjawu EP, Kio FE, Abu EK. Investigation of amplitude of accommodation among Ghanaian school children. Clin Exp Optom. 2012; 95(2):187-191.

6. PLOS authors have the option to publish the peer review history of their article ([what does this mean?](https://journals.plos.org/plosone/s/editorial-and-peer-review-process#loc-peer-review-history)). If published, this will include your full peer review and any attached files.

If you choose “no”, your identity will remain anonymous but your review may still be made public.

**Do you want your identity to be public for this peer review?** For information about this choice, including consent withdrawal, please see our [Privacy Policy](https://www.plos.org/privacy-policy).

Reviewer #1: No

Reviewer #2: No


[NOTE: If reviewer comments were submitted as an attachment file, they will be attached to this email and accessible via the submission site. Please log into your account, locate the manuscript record, and check for the action link "View Attachments". If this link does not appear, there are no attachment files to be viewed.]


While revising your submission, please upload your figure files to the Preflight Analysis and Conversion Engine (PACE) digital diagnostic tool, <https://pacev2.apexcovantage.com/>. PACE helps ensure that figures meet PLOS requirements. To use PACE, you must first register as a user. Registration is free. Then, login and navigate to the UPLOAD tab, where you will find detailed instructions on how to use the tool. If you encounter any issues or have any questions when using PACE, please email us at [figures@plos.org](mailto:figures@plos.org). Please note that Supporting Information files do not need this step.

- The end of response to reviewers’ comments –
